# Association Between Pharmacy Closures and Adherence to Cardiovascular Medications Among Older US Adults

**DOI:** 10.1001/jamanetworkopen.2019.2606

**Published:** 2019-04-19

**Authors:** Dima M. Qato, G. Caleb Alexander, Apurba Chakraborty, Jenny S. Guadamuz, John W. Jackson

**Affiliations:** 1 Division of Epidemiology and Biostatistics, School of Public Health, University of Illinois at Chicago; 2 Department of Pharmacy Systems, Outcomes and Policy, College of Pharmacy, University of Illinois at Chicago; 3 Department of Medicine, Johns Hopkins Medicine, Baltimore, Maryland; 4 Department of Epidemiology, Johns Hopkins Bloomberg School of Public Health, Baltimore, Maryland; 5 Center for Drug Safety and Effectiveness, Johns Hopkins Bloomberg School of Public Health, Baltimore, Maryland; 6 Institute of Minority Health Research, College of Medicine, University of Illinois at Chicago; 7 Department of Mental Health, Johns Hopkins Bloomberg School of Public Health, Baltimore, Maryland

## Abstract

**Importance:**

It is unknown whether and how pharmacy closures alter medication adherence.

**Objective:**

To examine the association between pharmacy closures and adherence to statins, β-blockers, and oral anticoagulants among adults 50 years or older in the United States.

**Design, Setting, and Participants:**

In this retrospective cohort study, comparative interrupted time series analyses were performed using a nationally representative 5% random sample of anonymized, longitudinal, individual-level pharmacy claims from IQVIA LRx LifeLink. Analyses included all prescription claims for individuals followed up between January 1, 2011, and December 31, 2016. Separate cohorts were derived for users of statins, β-blockers, and oral anticoagulants. The differential association of pharmacy closure was examined as a function of baseline adherence, pharmacy, and individual characteristics.

**Main Outcomes and Measures:**

Difference in monthly adherence, measured as proportion of days covered, during 12-month baseline and follow-up periods among patients using a pharmacy that subsequently closed (closure cohort) compared with their counterparts (control cohort).

**Results:**

Among 3 089 803 individuals filling at least 1 statin prescription between January 1, 2011, and December 31, 2016 (mean [SD] age, 66.3 [9.3] years; 52.0% female), 3.0% (n = 92 287) filled at a pharmacy that subsequently closed. Before closure, monthly adherence was similar in the closure and control cohorts (mean [SD], 70.5% [26.7%] vs 70.7% [26.5%]). In multivariable models, individuals filling at pharmacies that closed experienced an immediate and significant decline (on average, an absolute change of −5.90%; 95% CI, −6.12% to −5.69%) in statin adherence during the first 3 months after closure compared with their counterparts. This difference persisted over 12 months of follow-up. A similar decline in adherence was observed when examining cohorts using β-blockers (−5.71%; 95% CI, −5.96% to −5.46%) or oral anticoagulants (−5.63%; 95% CI, −6.24% to −5.01%). The mean association of pharmacy closure with adherence was greater among individuals using independent pharmacies (−7.89%; 95% CI, −8.32% to −7.47%) or living in neighborhoods with fewer pharmacies (−7.98%; 95% CI, −8.50% to −7.47%) compared with their counterparts.

**Conclusions and Relevance:**

Pharmacy closures are associated with persistent, clinically significant declines in adherence to cardiovascular medications among older adults in the United States. Efforts to reduce nonadherence to prescription medications should consider the role of pharmacy closures, especially among patients at highest risk.

## Key Points

**Question:**

What is the association between pharmacy closures and adherence to cardiovascular medications among US adults 50 years or older?

**Findings:**

Among 3.1 million individuals in this national cohort study, older adults filling statins, β-blockers, or oral anticoagulants at pharmacies that closed experienced an immediate statistically and clinically significant decline in adherence during the first 3 months after closure compared with their counterparts. This difference persisted over 12 months and was greater among older adults living in neighborhoods with fewer pharmacies.

**Meaning:**

Efforts to reduce nonadherence to prescription medications among older US adults should consider the role of pharmacy closures, especially among patients at highest risk.

## Teaser

This cohort study examines the association between pharmacy closures and adherence to statins, β-blockers, and oral anticoagulants among adults 50 years or older in the United States.

## Introduction

Nonadherence occurs in approximately 50% of patients taking prescription medications, including cardiovascular drugs like statins, β-blockers, and oral anticoagulants, which are widely used among older adults in the United States.^1,2,3^ Despite ongoing efforts to improve the affordability of prescription medications among older adults (eg, Medicare Part D^4^), nonadherence persists as an important public health problem. It is increasingly recognized that older adults may encounter system-level barriers to adherence beyond the high cost of prescription drugs.^5,6,7^

One such barrier is pharmacy accessibility. The geographic accessibility of pharmacies varies substantially across communities in the United States,^8,9^ and pharmacy closures may decrease pharmacy access and thereby limit patients’ ability to fill and adhere to prescribed medications. Such closures, which disproportionately influence independent pharmacies located in low-income urban and rural neighborhoods, have increased significantly since the implementation of Medicare Part D.^10^ Pharmacy closures are expected to further increase due to the expanding role of pharmacy benefit managers (PBMs) in the pharmacy market because of mergers and acquisitions^11,12^ and the growth of preferred pharmacy networks,^4^ which often exclude independent stores. Despite this trend, the association of pharmacy closure with adherence to prescription medications is not known.

We used nationally representative anonymized, longitudinal, individual-level pharmacy claims from IQVIA LRx LifeLink^13^ to quantify the consequences of pharmacy closures on adherence to statins, β-blockers, and oral anticoagulants among adults 50 years or older in the United States. We hypothesized that pharmacy closure would be associated with significant declines in adherence among older adults, even among those who had high adherence to their prescription medications at baseline.

## Methods

### Setting and Data

We used a nationally representative 5% random sample of individuals within IQVIA LRx LifeLink data followed up between January 1, 2011, and December 31, 2016. These anonymized, longitudinal, individual-level all-payer pharmacy claims are sampled from more than 240 million patients who fill prescription medications at retail pharmacies in the United States. The data are collected weekly from approximately 56 000 retail pharmacies in the United States (eTable 1 in the Supplement).^14^ The retail pharmacies captured in IQVIA LRx LifeLink data represent 97.1% (40 474 of 41 684) of all chains and include 15 074 of the 23 596 independent pharmacies (63.9%) located in the United States.^9^ In addition, individuals are continuously observed in these data because patients’ prescription fills are tracked across time and linked among retail and nonretail channels. Therefore, detailed prescription information is captured for all patients across all retail and nonretail pharmacies, including dispensing pharmacy type (chain, independent, mass merchandiser, or food), mail order (with days’ supply), method of payment (cash, Medicaid, Medicare Part D, or commercial third party), co-payment amount, and pharmacy location (5-digit zip code). This study was exempt from institutional review board approval because the University of Illinois at Chicago Office for the Protection of Research Subjects determined it did not constitute human participant research. This study followed the Strengthening the Reporting of Observational Studies in Epidemiology (STROBE) reporting guideline.

### Definition of Pharmacy Closure

We also used IQVIA LRx LifeLink data to identify retail pharmacies that closed during our study period. We defined pharmacy closures as pharmacies that were no longer in operation at a specific brick-and-mortar location; pharmacies that changed ownership were considered operational.

We also had an “active status” indicator for each pharmacy. Active status is defined by IQVIA as a pharmacy that was operational as of June 1, 2017 (the date the data were extracted). Therefore, we were able to differentiate whether the stoppage of reporting was an actual closure from a stoppage of reporting for pharmacies that remained operational (active).

The distribution of pharmacies overall and for pharmacies that permanently stopped reporting transactions to IQVIA by active status is included in eTable 2 in the Supplement. Of the 59 375 retail pharmacies reporting prescription transactions during our study period, 3622 (6.1%) had permanently stopped reporting prescription transactions to IQVIA at some point during our study period, were not operational (active), and were assumed to have closed. We defined the date of the last reported pharmacy transaction as the pharmacy closure date.

### Cohort Derivation

**Figure 1.**
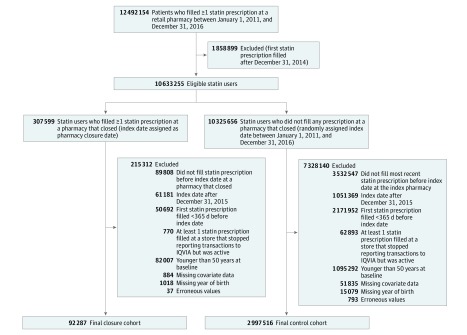
Cohort Selection for Statin Users ^a^An additional 884 patients with missing covariate data were excluded from the analysis. ^b^An additional 51 835 patients with missing covariate data were excluded from the analysis.

We focused on individuals using statins, β-blockers, or oral anticoagulants at any point during the study period and conducted 3 separate analyses, one for each therapeutic class of interest. For users of anticoagulants, we included both warfarin sodium and novel oral anticoagulants. Therefore, to examine the association of pharmacy closure with statin use, we first identified all individuals using a statin for a least 1 day during the study period (Figure 1). Next, we identified 307 599 statin users who filled a statin prescription at a pharmacy that subsequently closed. We defined these individuals as our closure cohort and all other statin users as our nonclosure (control) cohort.

We defined an index date for each individual to allow for baseline (12-month preclosure) and follow-up (12-month postclosure) assessment. Among the closure cohort, we defined the date of pharmacy closure as the index date and the pharmacy that closed as their index pharmacy. For the control cohort, we assigned a random date between January 1, 2011, and December 31, 2016, as the index date, and the pharmacy where individuals filled their most recent prescription directly before their index date was defined as their index pharmacy. Only individuals who filled the statin prescription directly before closure at their index pharmacy were included in our final cohort. Therefore, 89 808 of 307 599 (29.1%) and 3 532 547 of 10 325 656 (34.2%) individuals in the closure and control cohorts, respectively, who did not fill their most recent preindex statin prescription at their index pharmacy were excluded from the analysis. To avoid any potential misclassification, we also excluded individuals who filled at pharmacies that stopped reporting but were active.

To ensure 12-month baseline and follow-up periods, we excluded individuals in the closure and control cohorts who filled their first statin prescription less than 12 months before the index date, as well as individuals with an index date after December 31, 2015. Because we were interested in adults 50 years or older, we also excluded individuals younger than 50 years during their index year. Our final statin cohort included 92 287 individuals in the closure cohort and 2 997 516 individuals in the control cohort. We followed analogous procedures when examining the association of pharmacy closure with use of β-blockers and oral anticoagulants (eFigure 1 in the Supplement).

### Medication Adherence

Our primary outcome measure was medication adherence. For each individual, we measured adherence as the monthly proportion of days covered (PDC) for each month of their 12-month baseline (preclosure) and 12-month follow-up (postclosure) period. The PDC is a standard quality measure for adherence and is used by the Centers for Medicare & Medicaid Services as part of the Medicare Part D Five-Star Quality Rating System.^15,16^ It is defined as the percentage of days’ supply for a specific drug class, such as statins, over a specified interval. Using this definition, we calculated each individual’s PDC at each month (30-day interval) of their baseline and follow-up periods. To identify a subcohort of fully adherent individuals, we also classified individuals according to whether or not their 12-month PDC was at least 80% at baseline.

### Other Variables

We assessed pharmacy and individual characteristics during the baseline period. The focus was on variables that were either of substantial a priori interest or that have been previously associated with medication adherence.

#### Pharmacy Characteristics

We examined whether the associations of closure differed based on dispensing pharmacy type (chain, independent, mass merchandiser, or food) and location. For pharmacy location, we used the 2015 American Community Survey data to derive information on community demographics at the zip code level. We created a categorical variable to describe the predominant racial/ethnic composition of a community (eg, predominantly black communities were defined as those where ≥50% of the population is black). We categorized a zip code as low income if at least 20% of the population were below the federal poverty level. To quantify pharmacy access, we measured pharmacy density in the specific zip code of the index year as quintiles of the total number of pharmacies per square mile.

#### Individual Characteristics

We measured the extent to which the index pharmacy was used during the baseline period to ascertain individuals’ filling patterns. Specifically, we created a categorical variable based on the proportion of all prescriptions filled at the index pharmacy (<50%, 50%-99%, or 100%). In addition, we included a binary variable indicating whether a nonretail (eg, mail order) store was also used for at least 1 prescription for the drug class of interest during the baseline period. We hypothesized that pharmacy closure would have minimal repercussions on adherence among individuals using multiple stores.

For the last transaction during the baseline period, we characterized the method of payment (cash, Medicaid, Medicare Part D, or commercial third party) and co-payment amount ($0 to <$5, ≥$5 to <$10, or ≥$10). We also assessed individuals’ age and sex, as well as the presence of polypharmacy, which we defined as filling of 5 or more unique medications during the baseline period. Finally, we quantified the duration of statin use (in months) before the index date.

### Statistical Analysis

We performed a retrospective cohort study using a comparative interrupted time series design with a control group. To do so, we first plotted monthly adherence for our closure and control cohorts for each drug class at baseline (before closure) and during follow-up (after closure). We estimated propensity score weights to adjust for measured differences between the closure and control cohorts.^17^ We anticipated each person’s propensity score probability of being in the closure vs control cohort given the covariates listed in Table 1 using logistic regression. The weights were set to the propensity score odds for the control cohort and to 1 for the closure cohort. The weighted control cohort has the covariate distribution of the closure cohort, providing a counterfactual assumption for what would have happened under no pharmacy closure.

**Table 1.** Baseline Characteristics of Statin Cohort Participants^a^

Variable	Unweighted, %			Propensity Score Weighted, %		
	Total	Closure	Nonclosure	Total	Closure	Nonclosure
No. (%)	3 089 803 (100)	92 287 (3.0)	2 997 516 (97.0)	184 824 (100)	92 287 (49.9)	92 537 (50.1)
Age group, y						
≥50 to 64	45.1	42.1	45.2	42.1	42.1	42.1
≥65	54.9	57.9	54.8	57.9	57.9	57.9
Sex^b^						
Men	47.7	45.6	47.8	45.5	45.6	45.5
Women	52.0	53.1	52.0	53.0	53.1	53.0
Duration of statin use, mean (SD), mo^c^	30.2 (13.6)	29.6 (13.9)	30.2 (13.5)	29.5 (3.3)	29.6 (13.9)	29.5 (2.4)
Dispensing pharmacy type^d^						
Chain	50.8	21.1	51.8	21.0	21.1	21.0
Independent	11.7	27.8	11.2	27.9	27.8	28.1
Mass merchandiser	20.3	15.9	20.4	15.8	15.9	15.8
Food	17.2	35.3	16.6	35.2	35.3	35.1
Nonretail (eg, mail order)^e^						
Yes	4.8	4.0	4.8	4.0	4.0	4.0
No (retail only)	95.2	96.0	95.2	96.0	96.0	96.0
Share of prescriptions filled at index pharmacy^f^						
100% (index pharmacy only)	61.1	58.7	61.2	58.7	58.7	58.8
≥50% to ≤99%	32.3	35.2	32.2	35.2	35.2	35.2
<50%	6.6	6.1	6.6	6.1	6.1	6.1
Polypharmacy (filling of ≥5 unique medications)^g^						
Yes	89.7	91.3	89.6	91.3	91.3	91.3
No	10.4	8.7	10.4	8.7	8.7	8.7
Method of payment^h^						
Cash	4.7	5.3	4.7	5.3	5.3	5.3
Medicaid	3.3	4.0	3.3	4.1	4.0	4.1
Medicare Part D	43.4	43.7	43.4	43.7	43.7	43.7
Commercial third party	48.7	46.9	48.7	47.0	46.9	47.0
Co-payment amount^i^						
$0 to <$5	59.4	52.6	59.5	52.6	52.6	52.6
≥$5 to <$10	15.9	19.4	15.8	19.4	19.4	19.4
≥$10	24.7	28.0	24.6	28.0	28.0	28.0
Community type^j^						
White	76.6	72.1	76.7	72.1	72.1	72.1
Black	4.5	6.8	4.4	6.8	6.8	6.8
Hispanic/Latino	6.3	7.1	6.3	7.1	7.1	7.1
Diverse	12.0	13.5	11.9	13.5	13.5	13.5
Other	0.7	0.5	0.7	0.5	0.5	0.5
Urban/rural^k^						
Urban	28.9	35.1	28.7	35.1	35.1	35.2
Suburban	56.1	49.0	56.3	49.0	49.0	48.9
Rural	15.0	15.9	15.0	15.9	15.9	15.9
Low income^l^						
Yes	24.1	28.7	23.9	28.7	28.7	28.8
No	75.9	71.3	76.1	71.3	71.3	71.2
Pharmacy density (total No. of pharmacies per square mile)^m^						
Quintile 1 (<0.045)	20.0	18.5	20.0	18.5	18.5	18.5
Quintile 2 (≥0.045 to <0.158)	20.0	16.1	20.1	16.1	16.1	16.1
Quintile 3 (≥0.158 to <0.405)	20.0	18.7	20.0	18.7	18.7	18.7
Quintile 4 (≥0.405 to <0.820)	20.0	19.3	20.0	19.3	19.3	19.4
Quintile 5 (≥0.820)	20.0	27.3	19.8	27.3	27.3	27.4

Abbreviation: PDC, proportion of days covered.

^a^ Data source is IQVIA LRx LifeLink real-world data medical claims between January 1, 2011, and December 31, 2016.

^b^ Sex unknown for 1225 patients in the closure cohort and 8651 patients in the nonclosure cohort.

^c^ Duration of statin use from first statin prescription fill to last statin prescription fill before index statin transaction.

^d^ Retail pharmacies used for any prescription filled during baseline.

^e^ Use of mail-order pharmacies for statin prescription fill during baseline.

^f^ Share of all prescriptions filled at any retail store during baseline.

^g^ Number of unique medications filled during baseline.

^h^ Method of payment for index statin transaction. Medicaid includes dual-eligible Medicare/Medicaid plans, Fee for Service Medicaid, and Medicaid managed care.

^i^ Co-payment for index statin transaction.

^j^ Community type designated based on the predominant race/ethnicity of the population in the zip code of index pharmacy location according to the 2015 American Community Survey data from the US Census Bureau as follows: predominantly white community (≥50% population is white), predominantly black community (≥50% of the population is black), predominantly Hispanic/Latino community (≥50% of the population is Hispanic/Latino of any race), diverse (no race/ethnicity was ≥50%), or other.

^k^ Urban, suburban, and rural defined based on the 2015 American Community Survey data from the US Census Bureau as follows: urban (>3000 persons per square mile), suburban (1000-3000 persons per square mile), and rural (<1000 persons per square mile).

^l^ Low income defined as more than 20% of the population living in the zip code were below the federal poverty level.

^m^ Number of retail pharmacies per square mile for index year.

Using these weights, we fit separate patient-level segmented linear regression models for each drug class of interest.^18^ Our models included the following: a constant term, a linear time trend (which measured the preclosure or baseline trend), a binary indicator for exposure (closure vs control), and a binary indicator for the follow-up period (before vs after closure). We assessed pharmacy closure associations with an interaction term between exposure and the follow-up period. We used the coefficient of the interaction term between exposure (closure vs control) and event (variable to identify the months before and after the index date) to estimate the immediate change in the mean monthly PDC among patients experiencing pharmacy closure (thereby netting out the change among patients in the control cohort). The coefficient of the interaction term between cohort and trend was used to estimate the trend in monthly PDC after pharmacy closure among patients experiencing pharmacy closure (thereby netting out the trend among patients in the control cohort).

We controlled for correlated error terms with use of generalized estimating equations and normally distributed errors. We assumed that repeated observations of adherence over time for individual patients nested within the same index pharmacy followed an autoregressive correlation structure. We conducted similar analyses among a subcohort of individuals fully adherent (PDC ≥80%) at baseline. We then conducted a stratified analysis for each subgroup category (eg, dispensing pharmacy type) and included interaction terms in the models to test the heterogeneity of the association of pharmacy closure for each subgroup. Finally, we examined the proportion of patients discontinuing statins during follow-up, which we defined as having no refill for statins during the 12-month follow-up period.

We also conducted an exploratory analysis to determine whether the association of closure with adherence varied by expected refill date based on days’ supply for the last statin filled before the pharmacy closure. We hypothesized that adherence would decline most among individuals expected to refill their prescriptions shortly after (eg, ≤2 weeks) their pharmacy closes. Statistical significance was set at 2-sided *P* < .001.

### Sensitivity Analyses

While IQVIA captures data from almost all retail chains, some pharmacies, particularly independents, do not report their data to IQVIA. Therefore, some patients in the closure cohort may have started to fill their prescriptions at nonreporting pharmacies once their pharmacy closes. To address this potential nonrandom reporting bias, we conducted the following series of sensitivity analyses: (1) we restricted our statin cohort to individuals who continued to fill at least 1 prescription of any type during the follow-up period, (2) we restricted our statin cohort to statin users with at least 1 refill for statins during follow-up, (3) we excluded statin users who had filled at a closed store that lost all patients after closure (theoretically, the patients using these closed stores may have started to fill at a nonreporting pharmacy after closure and are thus not captured in IQVIA data), and (4) we restricted our statin cohort to individuals who filled all their prescriptions (including nonstatins) at stable pharmacies only. Our data include an annual “pharmacy stability” indicator for each pharmacy. Pharmacies that consistently provide data to IQVIA are defined as stable pharmacies. Although individuals filling at least 1 statin prescription at an active pharmacy that permanently stopped reporting data to IQVIA were already excluded from our cohorts, some individuals may have filled at pharmacies that inconsistently provide data to IQVIA (defined as unstable reporters). Such inconsistent observations could also theoretically bias our results.

## Results

### Pharmacy Closures

Between January 1, 2011, and December 31, 2016, we observed a total of 59 375 pharmacies, of which 3622 (6.1%) closed. Of these 3622 pharmacies, 41.9% were independent stores, and the remainder were food (27.3%), chain (15.6%), and mass merchandise (15.2%) stores.

### Study Population

Of 3 089 803 individuals filling at least 1 statin prescription between January 1, 2011, and December 31, 2016 (mean [SD] age, 66.3 [9.3] years; 52.0% female), 3.0% (n = 92 287) were in the closure cohort, and the remainder were in the control cohort (Figure 1). Compared with the control cohort, statin users in the closure cohort were significantly (*P* < .001 for all) more likely to fill their statins at pharmacies that were independent (27.8% vs 11.2%) and located in predominantly black (6.8% vs 4.4%), urban (35.1% vs 28.7%), and low-income (28.7% vs 23.9%) neighborhoods (Table 1). Similar patterns were observed among β-blocker and oral anticoagulant users (eTable 3 in the Supplement). However, these differences were no longer observed in the weighted cohorts.

### Pharmacy Closures and Medication Adherence

**Figure 2.**
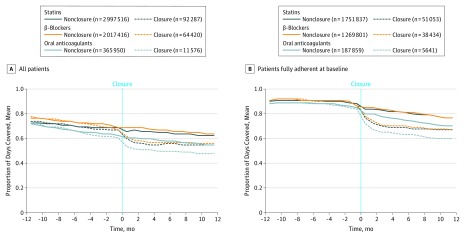
Pharmacy Closures and Medication Adherence *P* < .001 for all linear trends.

Before closure, there were no observed differences in adjusted monthly adherence between the statin closure and control cohorts (mean [SD], 70.5% [26.7%] vs 70.7% [26.5%]) (Figure 2). However, during the 12-month postclosure period, statin users in the closure cohort experienced, on average, an immediate and significant decline in statin adherence during the first 3 months after closure compared with their counterparts (absolute change, −5.90%; 95% CI, −6.12% to −5.69%) that persisted over 12 months of follow-up (Table 2). A similar decline in adherence was also observed among closure cohorts for β-blockers (−5.71%; 95% CI, −5.96% to −5.46%) and oral anticoagulants (−5.63%; 95% CI, −6.24% to −5.01%). For all 3 drug classes, individuals fully adherent (PDC ≥80%) at baseline also experienced clinically and statistically significant declines in adherence. These findings persist in our sensitivity analyses (eTable 4 in the Supplement).

**Table 2.** Association of Pharmacy Closure With Adherence to Statins, β-Blockers, and Oral Anticoagulants Overall and Among Patients Fully Adherent (PDC ≥80%) at Baseline^a^

Variable	Absolute % Change (95% CI)	
	Level^b^	Slope^c^
**All Patients**		
Statins	−5.90 (−6.12 to −5.69)	−0.15 (−0.18 to −0.13)
β-Blockers	−5.71 (−5.96 to −5.46)	−0.20 (−0.23 to −0.17)
Oral anticoagulants	−5.63 (−6.24 to −5.01)	−0.15 (−0.23 to −0.07)
**Patients Fully Adherent at Baseline**		
Statins	−8.57 (−8.84 to −8.30)	−0.18 (−0.22 to −0.15)
β-Blockers	−8.07 (−8.38 to −7.77)	−0.22 (−0.26 to −0.18)
Oral anticoagulants	−8.37 (−9.21 to −7.54)	−0.20 (−0.31 to −0.09)

Abbreviation: PDC, proportion of days covered.

^a^ Data source is IQVIA LRx LifeLink real-world data medical claims between January 1, 2011, and December 31, 2016. All data represent a statistically significant percentage change at *P* < .001.

^b^ Level refers to the immediate absolute decline (first 3 months) of the pharmacy closure on medication adherence.

^c^ Slope refers to the subsequent rate of change in adherence resulting from pharmacy closure.

The disproportionate decline in monthly adherence observed in the closure cohort was largely due to the complete discontinuation of medication rather than worsening or partial nonadherence (eFigure 2 in the Supplement). For example, among statin users, 23.8% in the closure cohort vs 12.8% in the control cohort (difference, 11.0%; 95% CI, 10.1%-11.9%) did not refill their statin prescriptions at any point during the 12-month follow-up period (eTable 5 in the Supplement). In addition, among statin users fully adherent at baseline, 15.3% in the closure cohort vs 3.5% in the control cohort (difference, 11.9%; 95% CI, 10.8%-12.9%) discontinued their statins.

### Pharmacy Closures and Medication Adherence Among Different Groups of Users

After adjustment and stratification by individual and pharmacy characteristics, a significant decline in adherence was observed among all subgroups in the closure cohort, although the magnitude of the decline varied significantly across groups. For example, among statin users, the decline in adherence was greater among individuals using independent pharmacies (−7.89%; 95% CI, −8.32% to −7.47%), filling all their prescriptions at a single store (−6.83%; 95% CI, −7.11% to −6.55%), or living in neighborhoods with fewer pharmacies (−7.98%; 95% CI, −8.50% to −7.47%) and was lowest among individuals also using nonretail channels, including mail order (−2.77%; 95% CI, −3.82% to −1.72%) (Table 3). Among individuals using chain stores or filling all their prescriptions at a single store, declines in adherence were even greater among those living in low-access neighborhoods with fewer pharmacies (eTable 6 in the Supplement).

**Table 3.** Association of Pharmacy Closure With Adherence to Statins Overall and Among Patients Fully Adherent at Baseline^a^

Variable	Absolute % Change, % (95% CI)			
	All Patients		Patients Fully Adherent at Baseline	
	Level	Slope	Level	Slope
Overall	−5.90 (−6.12 to −5.69)	−0.15 (−0.18 to −0.13)	−8.57 (−8.84 to −8.30)	−0.18 (−0.22 to −0.15)
Dispensing pharmacy type				
Chain	−6.89 (−7.34 to −6.43)	−0.21 (−0.27 to −0.15)	−9.07 (−9.61 to −8.52)	−0.26 (−0.33 to −0.18)
Independent	−7.89 (−8.32 to −7.47)	−0.26 (−0.31 to −0.20)	−10.91 (−11.47 to −10.36)	−0.31 (−0.38 to −0.24)
Mass merchandiser	−5.28 (−5.82 to −4.74)	−0.01 (−0.08 to 0.06)	−9.41 (−10.09 to −8.72)	−0.06 (−0.15 to 0.03)
Food	−3.97 (−4.33 to −3.62)	−0.12 (−0.17 to −0.07)	−5.98 (−6.41 to −5.55)	−0.13 (−0.19 to −0.07)
Nonretail (eg, mail order)				
Yes	−2.77 (−3.82 to −1.72)	0.06 (−0.08 to 0.20)	−3.15 (−4.35 to −1.95)	0.07 (−0.10 to 0.23)
No (retail only)	−6.03 (−6.25 to −5.81)	−0.16 (−0.19 to −0.13)	−8.82 (−9.10 to −8.55)	−0.20 (−0.23 to −0.16)
Share of prescriptions filled at index pharmacy				
100% (index pharmacy only)	−6.83 (−7.11 to −6.55)	−0.27 (−0.31 to −0.23)	−10.10 (−10.46 to −9.74)	−0.33 (−0.37 to −0.28)
≥50% to ≤99%	−4.77 (−5.13 to −4.41)	0.00 (−0.05 to 0.05)	−6.52 (−6.95 to −6.08)	0.02 (−0.04 to 0.07)
<50%	−3.40 (−4.26 to −2.54)	0.08 (−0.03 to 0.19)	−4.30 (−5.38 to −3.22)	0.02 (−0.13 to 0.17)
Polypharmacy (filling of ≥5 unique medications)				
Yes	−6.11 (−6.34 to −5.88)	−0.14 (−0.17 to −0.12)	−8.58 (−8.86 to −8.30)	−0.17 (−0.21 to −0.14)
No	−3.81 (−4.53 to −3.09)	−0.24 (−0.33 to −0.15)	−8.48 (−9.49 to −7.47)	−0.31 (−0.45 to −0.18)
Method of payment				
Cash	−4.04 (−4.97 to −3.10)	−0.39 (−0.51 to −0.26)	−5.74 (−6.98 to −5.49)	−0.42 (−0.59 to −0.25)
Medicaid	−8.11 (−9.22 to −7.00)	−0.04 (−0.18 to 0.10)	−12.81 (−14.43 to −11.19)	0.07 (−0.13 to 0.27)
Medicare Part D	−6.39 (−6.71 to −6.06)	−0.16 (−0.20 to −0.12)	−8.85 (−9.25 to −8.46)	−0.18 (−0.23 to −0.13)
Commercial third party	−5.49 (−5.81 to −5.18)	−0.13 (−0.17 to −0.09)	−8.27 (−8.66 to −7.87)	−0.18 (−0.24 to −0.13)
Co-payment amount				
$0 to <$5	−6.42 (−6.71 to −6.12)	−0.10 (−0.14 to −0.06)	−9.32 (−9.70 to −8.95)	−0.09 (−0.14 to −0.04)
≥$5 to <$10	−5.89 (−6.38 to −5.39)	−0.21 (−0.28 to −0.15)	−8.50 (−9.10 to −7.89)	−0.25 (−0.33 to −0.17)
≥$10	−4.92 (−5.32 to −4.52)	−0.21 (−0.27 to −0.16)	−7.16 (−7.66 to −6.66)	−0.31 (−0.38 to −0.25)
Community type				
White	−5.63 (−5.88 to −5.38)	−0.19 (−0.22 to −0.15)	−8.30 (−8.60 to −7.99)	−0.23 (−0.27 to −0.19)
Black	−6.68 (−7.55 to −5.81)	0.05 (−0.06 to 0.16)	−10.22 (−11.41 to −9.03)	0.18 (0.03 to 0.33)
Hispanic/Latino	−6.73 (−7.56 to −5.89)	−0.05 (−0.16 to 0.05)	−10.02 (−11.18 to −8.85)	−0.06 (−0.21 to 0.09)
Diverse	−6.57 (−7.17 to −5.98)	−0.11 (−0.19 to −0.04)	−8.70 (−9.46 to −7.95)	−0.13 (−0.23 to −0.03)
Other	−5.14 (−8.22 to −2.05)	−0.47 (−0.84 to −0.10)	−9.70 (−13.29 to −6.11)	−0.11 (−0.58 to 0.37)
Urban/rural				
Urban	−5.63 (−5.99 to −5.26)	−0.09 (−0.14 to −0.04)	−7.77 (−8.22 to −7.31)	−0.07 (−0.13 to −0.01)
Suburban	−5.45 (−5.76 to −5.15)	−0.13 (−0.17 to −0.09)	−8.15 (−8.52 to −7.77)	−0.17 (−0.22 to −0.12)
Rural	−7.87 (−8.42 to −7.32)	−0.37 (−0.44 to −0.30)	−11.42 (−12.11 to −10.72)	−0.47 (−0.56 to −0.38)
Low income				
Yes	−6.56 (−6.97 to −6.14)	−0.06 (−0.11 to −0.00)	−9.47 (−10.01 to −8.93)	−0.07 (−0.14 to −0.00)
No	−5.65 (−5.90 to −5.39)	−0.19 (−0.23 to −0.16)	−8.24 (−8.55 to −7.93)	−0.22 (−0.26 to −0.18)
Pharmacy density (total No. of pharmacies per square mile)				
Quintile 1 (<0.045)	−7.98 (−8.50 to −7.47)	−0.36 (−0.43 to −0.30)	−11.81 (−12.46 to −11.15)	−0.46 (−0.54 to −0.37)
Quintile 2 (≥0.045 to <0.158)	−5.91 (−6.44 to 5.38)	−0.09 (−0.16 to −0.02)	−8.70 (−9.35 to −8.04)	−0.11 (−0.20 to −0.03)
Quintile 3 (≥0.158 to <0.405)	−5.20 (−5.68 to −4.71)	−0.08 (−0.14 to −0.02)	−7.39 (−7.98 to −6.81)	−0.11 (−0.19 to −0.03)
Quintile 4 (≥0.405 to <0.820)	−5.06 (−5.54 to −4.57)	−0.17 (−0.23 to −0.10)	−7.44 (−8.04 to −6.85)	−0.18 (−0.27 to −0.10)
Quintile 5 (≥0.820)	−5.59 (−6.01 to −5.17)	−0.10 (−0.15 to −0.04)	−7.78 (−8.31 to −7.25)	−0.09 (−0.16 to −0.02)

^a^ Data source is IQVIA LRx LifeLink real-world data medical claims between January 1, 2011, and December 31, 2016. Interaction terms in the linear regression models used to assess the heterogeneity of the association of pharmacy closure vs nonclosure with monthly proportion of days covered for statins among each subgroup analyzed were statistically significant (*P* < .001) for all covariates overall and among patients fully adherent at baseline. Level refers to the immediate absolute decline (first 3 months) of the pharmacy closure on medication adherence. Slope refers to the subsequent rate of change in adherence resulting from pharmacy closure.

Although the association between closures and decline in adherence did not appear to vary substantially by co-payment amount or by community characteristics, such as income levels, declines in adherence were greater among individuals with Medicaid coverage (−8.11; 95% CI, −9.22 to −7.00) (Table 3). Similar patterns were observed among users of β-blockers and oral anticoagulants (eTable 7 in the Supplement). According to our exploratory analyses, declines in adherence were highest in statin users with a refill expected within 14 days after pharmacy closure (−14.67%; 95% CI, −15.18% to −14.16%) and lowest if a refill was due more than 30 days after pharmacy closure (−1.91%; 95% CI, −2.24% to −1.58%) (eTable 8 in the Supplement).

## Discussion

Despite the critical role of pharmacies in the supply and provision of prescription drugs to most American individuals, it is unknown whether and how pharmacy closure alters medication adherence. Using anonymized, longitudinal, individual-level pharmacy claims, we found that pharmacy closures are associated with clinically and statistically significant reductions in adherence to essential cardiovascular medications among older adults in the United States. Declines in adherence were most pronounced among older adults using independent pharmacies, purchasing from a single store to fill all their prescriptions, or living in low-access neighborhoods with fewer pharmacies and were consistent across several classes of cardiovascular medications. Our findings underscore the substantial influence of system-level factors beyond the high cost of prescription drugs on medication nonadherence, especially among patients at highest risk.

The reductions in adherence from pharmacy closure that we document are of a similar magnitude as gains that have been observed with adherence interventions designed to improve the affordability of prescription drugs.^6,19,20^ For example, eliminating co-payments for cardiovascular medicines among patients with a recent myocardial infarction improved adherence to statins and β-blockers by 7% to 10%.^20^ Similarly, value-based insurance designs, including low cost-sharing, have been demonstrated to increase adherence by 5% to 6%,^6^ while preferred networks may improve adherence by 1% to 2%.^19,21^ Although these studies do not represent a comprehensive assessment of interventions to promote adherence, they nevertheless underscore the magnitude and importance of our findings.

The association of pharmacy closure with nonadherence was mitigated but not fully abated among older adults using retail chains rather than independent pharmacies. Although chains may implement integrated management information systems and be better equipped to electronically transfer prescriptions from a closed store to another pharmacy within their organization, these transfer stores may be located farther away and be less accessible. In fact, in low-access neighborhoods, we found that declines in adherence after store closure are larger among patients using chains than independent pharmacies, which we speculate could be because chains may be less prevalent than independent stores among low-access neighborhoods.^8^

Several policies can mitigate the potential deleterious association of pharmacy closure with patients’ access to medications and health outcomes. Policies that ensure sufficient pharmacy reimbursement for prescription medications may diminish the number of at-risk pharmacies from closing. Since the implementation of Medicare Part D in 2006, low reimbursement rates by PBMs have often been cited as an underlying cause of pharmacy closures.^10^ In addition, provisions to increase the number and stability of preferred pharmacy networks, which often exclude many local (largely independent) pharmacies,^22,23^ are also important. Although preferred pharmacy networks attempt to improve adherence by encouraging patients to use a single store or “pharmacy home” for a lower co-payment,^24,25^ our results suggest that adherence declines most when an individual’s pharmacy home closes. Therefore, existing Centers for Medicare & Medicaid Services regulations that require participating Medicare Part D plans to meet convenient pharmacy access standards^26^ could also mandate minimum standards for pharmacy reimbursement for participating plans and their PBMs^27^ and include specific provisions that mandate similar access standards for preferred pharmacies that ensure their stability.

Alongside these policy changes, several strategies might directly target patients most at risk for experiencing a pharmacy closure. Such interventions include direct outreach to patients using pharmacies located in low-income urban neighborhoods disproportionately influenced by pharmacy closure, conversion by health plans to an open pharmacy network to permit greater flexibility regarding where prescriptions can be filled, or use of mail order to offset potential access barriers. These interventions are supported by our findings that individuals using mail order or filling at multiple pharmacies were least influenced when one of their pharmacies closes. Providing transportation services to and from patients’ pharmacy of choice^28^ may also be helpful for some select populations, particularly those with Medicaid coverage,^29^ as would increasing patient awareness of planned pharmacy closures and incorporating closure dates into refill management programs.^30^

### Strengths and Limitations

To our knowledge, this is the first study to examine the influence of pharmacy closures on adherence to prescription medications. The study is national in scope, with a large sample size, and uses all-payer pharmacy claims, which capture prescription data in both retail and nonretail pharmacies, including mail order. This study also used a strong quasi-experimental study design with a control group. IQVIA, which captures prescription data for more than 85% of all retail pharmacies in the United States, is the most optimal data source for these analyses. In addition, our findings persist in a series of sensitivity analyses.

Despite these strengths, our study has several limitations. First, experiencing a closure could be correlated with several unobserved factors that could also influence adherence. However, we attempted to mitigate this concern by incorporating weighted controls that share similar characteristics and similar preclosure trends. Second, our data are limited to patients who filled at least 1 prescription at a retail pharmacy in the United States. Therefore, our analyses may underestimate the association of pharmacy closures with medication adherence because our cohorts do not include patients who may have experienced primary nonadherence. Third, our data capture two-thirds of all independent pharmacies. Therefore, our findings may underestimate not only the closure of independents but also pharmacy access (and the associations of closure with adherence) in communities where these pharmacies are located. Fourth, measures of community characteristics are based on pharmacy location and not patients’ place of residence. Therefore, we may underestimate the association of closure in communities that lack pharmacies. Fifth, we did not examine the health outcomes that may be associated with the change in adherence we document.

## Conclusions

To our knowledge, this is the first assessment of the association of pharmacy closures with medication adherence. We found that pharmacy closures are associated with persistent and clinically significant declines in adherence to cardiovascular medications among older adults in the United States. Efforts aimed at reducing barriers to prescription medication adherence should consider the role of pharmacy closures, especially in patients at highest risk.

## Notes

Supplement. **eTable 1.** Total Number of Unique Pharmacies by Store Type for Retail and Non-retail Channels in IQVIA LRx National Sample (January 1, 2011-December 31, 2016) **eTable 2.** Distribution of Pharmacies by Stoppage of Reporting and Active Status in IQVIA LifeLink LRx Between January 1, 2011-December 31, 2016 **eTable 3.** Baseline Characteristics of β-Blocker and Oral Anticoagulant Users **eTable 4.** Sensitivity Analysis for Statin Users **eTable 5.** Discontinuation to Statins, β-Blockers, and Oral Anticoagulants Following Closure of Index Store Among Older Adults in the US **eTable 6.** Impact of Pharmacy Closures on Statin Adherence Stratified by Select Patient Subgroups Stratified by Pharmacy Access Quintiles **eTable 7.** Impact of Pharmacy Closures on β-Blocker and Oral Anticoagulant Adherence Stratified by Patient Subgroups **eTable 8.** Effect of Pharmacy Closures on Statin Adherence Stratified by Patient Subgroups Overall and Among Patients Fully Adherent at Baseline **eFigure 1.** Cohort Selection for β-Blocker and Oral Anticoagulant Users **eFigure 2.** Distribution of Proportion of Days Covered (PDC) During Baseline and Follow-up
