# Supplementary material for: Association Between Pharmacy Closures and Adherence to Cardiovascular Medications Among Older US Adults
Source: JAMA Netw Open. 2019 Apr 19;2(4):e192606. doi: 10.1001/jamanetworkopen.2019.2606 (PMC6481442; doi:10.1001/jamanetworkopen.2019.2606)
Supplement: Supplement. — eTable 1. Total Number of Unique Pharmacies by Store Type for Retail and Non-retail Channels in IQVIA LRx National Sample (January 1, 2011-December 31, 2016) eTable 2. Distribution of Pharmacies by Stoppage of Reporting and Active Status in IQVIA LifeLink LRx Between January 1, 2011-December 31, 2016 eTable 3. Baseline Characteristics of β-Blocker and Oral Anticoagulant Users eTable 4. Sensitivity Analysis for Statin Users eTable 5. Discontinuation to Statins, β-Blockers, and Oral Anticoagulants Following Closure of Index Store Among Older Adults in the US eTable 6. Impact of Pharmacy Closures on Statin Adherence Stratified by Select Patient Subgroups Stratified by Pharmacy Access Quintiles eTable 7. Impact of Pharmacy Closures on β-Blocker and Oral Anticoagulant Adherence Stratified by Patient Subgroups eTable 8. Effect of Pharmacy Closures on Statin Adherence Stratified by Patient Subgroups Overall and Among Patients Fully Adherent at Baseline eFigure 1. Cohort Selection for β-Blocker and Oral Anticoagulant Users eFigure 2. Distribution of Proportion of Days Covered (PDC) During Baseline and Follow-up [file jamanetwopen-2-e192606-s001.pdf]

# Supplementary Online Content

Qato DM, Alexander GC, Chakraborty A, Guadamuz JS, Jackson JW. Association between pharmacy closures and adherence to cardiovascular medications among older US adults. *JAMA Netw Open*. 2019;2(4):e192606. doi:10.1001/jamanetworkopen.2019.2606

**eTable 1.** Total Number of Unique Pharmacies by Store Type for Retail and Non-retail Channels in IQVIA LRx National Sample (January 1, 2011-December 31, 2016)

**eTable 2.** Distribution of Pharmacies by Stoppage of Reporting and Active Status in IQVIA LifeLink LRx Between January 1, 2011-December 3, 2016

**eTable 3.** Baseline Characteristics of β-Blocker and Oral Anticoagulant Users

**eTable 4.** Sensitivity Analysis for Statin Users

**eTable 5.** Discontinuation to Statins, β-Blockers, and Oral Anticoagulants Following Closure of Index Store Among Older Adults in the US

**eTable 6.** Impact of Pharmacy Closures on Statin Adherence Stratified by Select Patient Subgroups Stratified by Pharmacy Access Quintiles

**eTable 7.** Impact of Pharmacy Closures on β-Blocker and Oral Anticoagulant Adherence Stratified by Patient Subgroups

**eTable 8.** Effect of Pharmacy Closures on Statin Adherence Stratified by Patient Subgroups Overall and Among Patients Fully Adherent at Baseline

**eFigure 1.** Cohort Selection for β-Blocker and Oral Anticoagulant Users

**eFigure 2.** Distribution of Proportion of Days Covered (PDC) During Baseline and Follow-up

This supplementary material has been provided by the authors to give readers additional information about their work.

## Online Supplement

**eTable 1.** Total number of unique pharmacies by store type for retail and non-retail channels in IQVIA LRx National Sample (January 1st 2011-December 31st 2016)

| | 2011 | 2012 | 2013 | 2014 | 2015 | 2016 |
|---|---|---|---|---|---|---|
| Overall | 50,828 (100%) | 52,094 (100%) | 58,613 (100%) | 59,746 (100%) | 59,823 (100%) | 60,379 (100%) |
| Retail | 48,088 (94.6%) | 49,322 (94.7%) | 53,988 (92.1%) | 55,042 (92.1%) | 55,147 (92.2%) | 55,548 (92.0%) |
| Chains[1] | 38,181 (79.4%) | 39,203 (79.5%) | 39,837 (74.8%) | 40,282 (73.2%) | 40,400 (73.2%) | 40,474 (72.9%) |
| Independent | 9,907 (19.5) | 10,119 (19.4%) | 14,151 (24.1) | 14,760 (26.8%) | 14,747 (26.7%) | 15,074 (27.1%) |
| Non-Retail[2] | 2,740 (5.4%) | 2,758 (5.3%) | 4,611 (7.9%) | 4,693 (7.9%) | 4,663 (7.8%) | 4,820 (8.0%) |

[1]Includes pharmacies located in food and mass merchandise stores;[2]Includes mail-service and long-term care pharmacies.

© 2019 Qato DM et al. *JAMA Network Open*.

**eTable 2**. Distribution of Pharmacies by Stoppage of Reporting and Active Status in IQVIA LifeLink LRx between January 1st, 2011-December 31st, 2016

| | Overall | Chains | Independents | Mass | Food |
|---|---|---|---|---|---|
| Total No. of Pharmacies | 59,375 (100%) | 22,724 (100%) | 16,981 (100%) | 8,912 (100%) | 10,758 (100%) |
| Stopped Reporting, Active | 2,332 (3.9%) | 306 (1.3%) | 1,570 (9.2%) | 102 (1.1%) | 354 (3.3%) |
| **Stopped Reporting, Inactive*** | 3,622 (6.1%) | 564 (2.5%) | 1,518 (8.9%) | 551 (6.2%) | 989 (9.2%) |
| Active and/or did not stop reporting | 53,421 (90%) | 21,854 (96%) | 13,893 (82%) | 8,259 (93%) | 9,415 (86%) |

* Stores that permanently stopped reporting and closed..

© 2019 Qato DM et al. *JAMA Network Open*.

**eTable 3. Baseline Characteristics of β-Blocker and Oral Anticoagulant Users**

**A. Baseline characteristics of β-blocker users**

| | Characteristics, % | | | | | |
|---|---|---|---|---|---|---|
| | Unweighted | | | Propensity Score Weighted | | |
| | Total | Closure | Non-Closure | Total | Closure | Non-Closure |
| Participants, n (%) | 2,081,836 (100%) | 64,420 (3.1%) | 2,017,416 (96.9%) | 129,970 (100%) | 64,420 (49.9%) | 64,550 (50.1%) |
| Age group, years | | | | | | |
| ≥50 to 64 | 40.4 | 37.3 | 40.5 | 37.3 | 37.3 | 37.3 |
| ≥65 | 59.6 | 62.7 | 59.5 | 62.7 | 62.7 | 62.7 |
| Gender[a] | | | | | | |
| Women | 54.6 | 55.4 | 54.5 | 55.4 | 55.4 | 55.4 |
| Men | 45.1 | 43.3 | 45.1 | 43.2 | 43.3 | 43.2 |
| Duration of beta blocker use in months, mean (SD)[b] | 30.0 (13.5) | 29.5 (13.9) | 30.1 (13.5) | 29.5 (3.4) | 29.5 (13.9) | 29.4 (2.4) |
| Index pharmacy type | | | | | | |
| Chain | 49.4 | 20.8 | 50.3 | 20.8 | 20.8 | 20.8 |
| Independent | 12.5 | 28.5 | 12.0 | 28.7 | 28.5 | 28.8 |
| Mass merchandiser | 21.0 | 16.1 | 21.2 | 16.1 | 16.1 | 16.1 |
| Food | 17.1 | 34.5 | 16.6 | 34.4 | 34.5 | 34.4 |
| Mail-order[d] | | | | | | |
| Yes | 4.8 | 4.2 | 4.9 | 4.1 | 4.2 | 4.0 |
| No | 95.2 | 95.9 | 95.1 | 95.9 | 95.9 | 95.0 |
| Share of prescriptions filled at index pharmacy[e] | | | | | | |
| 100% | 60.5 | 58.5 | 60.6 | 58.5 | 58.5 | 58.6 |
| ≥50% to <99% | 32.6 | 35.6 | 32.5 | 35.5 | 35.6 | 35.5 |
| <50% | 6.9 | 5.9 | 7.0 | 5.9 | 5.9 | 6.0 |
| Polypharmacy (use of ≥ 5 medications)[f] | | | | | | |
| Yes | 91.7 | 93.1 | 91.7 | 93.1 | 93.1 | 93.1 |
| No | 8.3 | 6.9 | 8.3 | 6.9 | 6.9 | 6.9 |
| Method of Payment[g] | | | | | | |
| Cash | 6.8 | 6.1 | 6.8 | 6.0 | 6.1 | 6.0 |
| Medicaid | 3.4 | 4.1 | 3.4 | 4.1 | 4.1 | 4.2 |
| Part D | 46.1 | 46.9 | 46.1 | 46.9 | 46.9 | 46.9 |
| Third party | 43.7 | 42.9 | 43.7 | 42.9 | 42.9 | 43.0 |
| Copayment[h] | | | | | | |
| $0 to <$5 | 66.0 | 59.8 | 66.2 | 59.8 | 59.8 | 59.7 |
| ≥$5 to <$10 | 16.8 | 19.5 | 16.8 | 19.5 | 19.5 | 19.4 |
| ≥$10 | 17.2 | 20.8 | 17.0 | 20.8 | 20.8 | 20.8 |
| Community type[i] | | | | | | |
| White | 77.2 | 73.0 | 77.3 | 73.0 | 73.0 | 73.0 |
| Black | 4.8 | 7.3 | 4.8 | 7.3 | 7.3 | 7.3 |
| Hispanic/Latino | 5.9 | 6.4 | 5.9 | 6.4 | 6.4 | 6.4 |
| Diverse | 11.5 | 13.0 | 11.5 | 12.9 | 13.0 | 12.9 |
| Other | 0.5 | 0.4 | 0.5 | 0.4 | 0.4 | 0.4 |
| Urbanity[j] | | | | | | |
| Urban | 27.7 | 34.3 | 27.5 | 34.3 | 34.3 | 34.3 |
| Suburban | 55.3 | 48.0 | 55.5 | 48.0 | 48.0 | 47.9 |
| Rural | 17.1 | 17.7 | 17.1 | 17.7 | 17.7 | 17.7 |
| Low Income[k] | | | | | | |
| Yes | 25.3 | 29.4 | 25.2 | 29.4 | 29.4 | 29.5 |
| No | 74.7 | 70.7 | 74.8 | 70.6 | 70.7 | 70.5 |
| Pharmacy Density (per sq. mile)[l] | | | | | | |
| Quintile 1 (<0.039) | 20.0 | 18.1 | 20.1 | 18.1 | 18.1 | 18.1 |
| Quintile 5 (≥0.799) | 20.0 | 27.5 | 19.8 | 27.5 | 27.5 | 27.5 |
| Mean Monthly PDC, Mean (SD) | 0.74 (0.26) | 0.73 (0.26) | 0.74 (0.26) | 0.73 (0.06) | 0.73 (0.26) | 0.74 (0.05) |

© 2019 Qato DM et al. *JAMA Network Open*.

## B. Baseline characteristics of oral anticoagulant users

| | Characteristics, % | | | | | |
|---|---|---|---|---|---|---|
| | Unweighted | | | Propensity Score Weighted | | |
| | Total | Closure | Non-Closure | Total | Closure | Non-Closure |
| Participants, n (%) | 377,526 (100%) | 11,576 (3.1%) | 365,950 (96.9%) | 23,177 (100%) | 11,576 (49.9%) | 11,601 (50.1%) |
| Age group, years | | | | | | |
| ≥50 to 64 | 24.9 | 22.8 | 24.9 | 22.8 | 22.8 | 22.8 |
| ≥65 | 75.1 | 77.2 | 75.1 | 77.2 | 77.2 | 77.2 |
| Gender[a] | | | | | | |
| Women | 46.5 | 47.4 | 46.5 | 47.3 | 47.4 | 47.3 |
| Men | 53.1 | 51.3 | 53.2 | 51.3 | 51.3 | 51.2 |
| Duration of oral anticoagulant use in months, mean (SD)[b] | 28.1 (13.6) | 27.3 (13.8) | 28.1 (13.6) | 27.3 (3.4) | 27.3 (13.8) | 27.3 (2.4) |
| Index pharmacy type | | | | | | |
| Chain | 49.3 | 19.5 | 50.3 | 19.5 | 19.5 | 19.4 |
| Independent | 13.0 | 29.7 | 12.4 | 29.8 | 29.7 | 30.0 |
| Mass merchandiser | 20.0 | 16.0 | 20.1 | 16.0 | 16.0 | 16.0 |
| Food | 17.8 | 34.8 | 17.2 | 34.7 | 34.8 | 34.7 |
| Mail-order[d] | | | | | | |
| Yes | 5.4 | 4.4 | 5.4 | 4.5 | 4.4 | 4.5 |
| No | 94.6 | 95.6 | 94.6 | 95.6 | 95.6 | 95.5 |
| Share of prescriptions filled at index pharmacy[e] | | | | | | |
| 100% | 58.2 | 56.2 | 58.3 | 56.2 | 56.2 | 56.3 |
| ≥50% to <99% | 34.6 | 37.5 | 34.5 | 37.5 | 37.5 | 37.4 |
| <50% | 7.2 | 6.3 | 7.2 | 6.3 | 6.3 | 6.3 |
| Polypharmacy (use of ≥ 5 medications)[f] | | | | | | |
| Yes | 94.7 | 95.7 | 94.7 | 95.7 | 95.7 | 95.7 |
| No | 5.3 | 4.3 | 5.3 | 4.3 | 4.3 | 4.3 |
| Method of Payment[g] | | | | | | |
| Cash | 5.9 | 5.2 | 5.9 | 5.2 | 5.2 | 5.1 |
| Medicaid | 2.2 | 2.3 | 2.2 | 2.3 | 2.3 | 2.4 |
| Part D | 54.2 | 53.5 | 54.2 | 53.5 | 53.5 | 53.5 |
| Third party | 37.8 | 39.0 | 37.7 | 39.0 | 39.0 | 39.0 |
| Copayment[h] | | | | | | |
| $0 to <$5 | 60.0 | 51.1 | 60.3 | 51.1 | 51.1 | 51.1 |
| ≥$5 to <$10 | 16.7 | 20.8 | 16.6 | 20.8 | 20.8 | 20.8 |
| ≥$10 | 23.3 | 28.1 | 23.2 | 28.1 | 28.1 | 28.1 |
| Community type[i] | | | | | | |
| White | 81.8 | 79.1 | 81.9 | 79.1 | 79.1 | 79.1 |
| Black | 3.6 | 4.9 | 3.6 | 5.0 | 4.9 | 5.0 |
| Hispanic/Latino | 4.3 | 4.6 | 4.3 | 4.6 | 4.6 | 4.6 |
| Diverse | 9.9 | 11.1 | 9.8 | 11.0 | 11.1 | 11.0 |
| Other | 0.4 | 0.4 | 0.4 | 0.4 | 0.4 | 0.4 |
| Urban-rural[j] | | | | | | |
| Urban | 25.5 | 31.3 | 25.3 | 31.3 | 31.3 | 31.3 |
| Suburban | 57.1 | 50.2 | 57.3 | 50.2 | 50.2 | 50.2 |
| Rural | 17.4 | 18.5 | 17.4 | 18.5 | 18.5 | 18.6 |
| Low Income[k] | | | | | | |
| Yes | 25.3 | 25.5 | 22.4 | 25.6 | 25.5 | 25.6 |
| No | 74.7 | 74.5 | 77.6 | 74.4 | 74.5 | 74.4 |
| Pharmacy Density (per sq. mile)[l] | | | | | | |
| Quintile 1 (<0.039) | 20.0 | 19.1 | 20.0 | 19.1 | 19.1 | 19.1 |
| Quintile 5 (≥0.750) | 20.0 | 27.1 | 19.8 | 27.1 | 27.1 | 27.2 |

## eTable 4. Sensitivity analysis for statin users

| | All Patients | | Patients Fully Adherent at Baseline | |
|---|---|---|---|---|
| | Percent Change, % (95% CI) | | Percent Change, % (95% CI) | |
| | Level[a] | Slope[b] | Level[a] | Slope[b] |
| At least one prescription during follow-up | -4.27 (-4.49 to -4.04) | 0.18 (0.15 - 0.21) | -5.64 (-6.70 to -4.59) | 0.41 (0.27 to 0.56) |
| At least one statin prescription during follow-up | -3.88 (-4.11 to -3.65) | 0.38 (0.35 to 0.41) | -4.96 (-5.21 to -4.71) | 0.37 (0.33 to 0.40) |
| Excluding patients that filled at closed pharmacies that are potential non-reporters[1] | -5.59 (-5.81 to -5.37) | -0.10 (-0.13 to -0.07) | -8.10 (-8.36 to -7.83) | -0.12 (-0.16 to -0.09) |
| All prescriptions filled at stable pharmacies only[2] | -6.30 (-7.11 to -5.50) | -0.30 (-0.42 to 0.19) | -13.36 (-14.89 to -11.82) | -0.27 (-0.48 to -0.06) |

[1] Pharmacies that are potential non-reporters lost all their paitents (of those included in LifeLink LRx sample) post-closure. Only 47 stores or 1.3% of pharmacies that closed are potential non-reporters and 1,469 or 1.6% of the 92,287 individuals in the statin cohort filled at these stores.
[2]Stable pharmacies are pharmacies that consistently supply data to IQVIA without any disruption, including transient (vs. permanent) disruption. In order to be flagged as a 'stable' reporter for the year, a pharmacy's reported prescription volume for each month of the year needs to be at least 50% of the average reported volume for the prior 10 weeks.

**eTable 5. Discontinuation to statins, β-blockers, and oral anticoagulants following closure of index store among older adults in the US**

| | | All Patients | | Patients fully adherent during baseline | |
|---|---|---|---|---|---|
| | | % | PR (95% CI) | % | PR (95% CI) |
| Statins | | | | | |
| Non-closure | | 12.8 | Reference | 3.5 | Reference |
| Closure | | 23.8 | 1.86 (1.83-1.90)*** | 15.3 | 4.39 (4.18-4.61)*** |
| β-blockers | | | | | |
| Non-closure | | 13.1 | Reference | 3.8 | Reference |
| Closure | | 24.2 | 1.85 (1.80-1.89)*** | 15.2 | 4.00 (3.79-4.23)*** |
| Oral anticoagulants | | | | | |
| Non-closure | | 19.0 | Reference | 5.4 | Reference |
| Closure | | 28.3 | 1.50 (1.43-1.57)*** | 16.9 | 3.15 (2.79-3.56)*** |

Abbreviations: PR, prevalence ratio; CI, confidence interval.
*** Statistically significant percent change at *P*-value<0.001.

© 2019 Qato DM et al. *JAMA Network Open*.

**eTable 6**. Impact of pharmacy closures on statin adherence stratified by select patient subgroups stratified by pharmacy access quintiles

| | **Absolute Percent Change, % (95% CI)** | | **Absolute Percent Change, % (95% CI)** | |
|---|---|---|---|---|
| | **Lowest access quintile** | | **Highest access quintile** | |
| | **Level[a]** | **Slope[b]** | **Level[a]** | **Slope[b]** |
| **Overall** | -7.98 (-8.50 to -7.47) | -0.36 (-0.43 to -0.30) | -5.59 (-6.01 to -5.17) | -0.10 (-0.15 to -0.04) |
| **Index pharmacy type** | | | | |
| Chain | -11.06 (-12.40 to -9.73) | -0.92 (-1.09 to -0.95) | -5.60 (-6.41 to -4.80) | 0.13 (0.02 to 0.23) |
| Independent | -7.81 (-8.52 to -7.09) | -0.34 (-0.43 to -0.25) | -10.00 (-10.89 to -9.10) | -0.36 (-0.47 to -0.25) |
| Mass merchandiser | -6.31 (-7.46 to -5.16) | -0.13 (-0.28 to 0.02) | -5.32 (-6.82 to -3.82) | 0.32 (0.13 to 0.52) |
| Food | -7.57 (-8.91 to -6.24) | -0.17 (-0.34 to 0.00) | -3.26 (-3.90 to -2.62) | -0.17 (-0.25 to -0.08) |
| **Share of all prescriptions filled at index pharmacy** | | | | |
| 100% | -9.79 (-10.40 to -9.09) | -0.57 (-0.66 to -0.49) | -6.21 (-6.76 to -5.66) | -0.20 (-0.27 to -0.17) |
| ≥50% to <99% | -5.63 (-6.49 to -4.77) | -0.02 (-0.13 to 0.09) | -5.01 (-5.73 to -4.30) | -0.03 (-0.07 to 0.12) |
| <50% | -1.07 (-3.46 to 1.33) | -0.03 (-0.32 to 0.26) | -3.05 (-4.66 to -1.44) | 0.14 (-0.07 to 0.35) |
| **Method of Payment** | | | | |
| Cash | -5.48 (-7.67 to -3.30) | -0.54 (-0.83 to -0.25) | -3.17 (-4.89 to -1.46) | -0.45 (-0.68 to -0.22) |
| Medicaid | -10.97 (-13.77 to -8.18) | -0.23 (-058 to 0.13) | -8.97 (-10.74 to -7.21) | -0.05 (-0.27 to 0.17) |
| Medicare part D | -8.54 (-9.28 to -7.80) | -0.36 (-0.46 to -0.27) | -5.96 (-6.58 to -5.34) | -0.16 (-0.24 to -0.08) |
| Third party | -7.46 (-8.24 to -6.68) | -0.35 (-0.45 to -0.25) | -5.03 (-5.67 to -4.38) | 0.02 (-0.06 to 0.10) |
| **Copayment** | | | | |
| $0 to <$5 | -8.75 (-9.47 to -8.03) | -0.35 (-0.44 to -0.26) | -6.24 (-6.79 to -5.69) | -0.11 (-0.18 to -0.04) |
| ≥$5 to <$10 | -7.90 (-9.05 to -6.74) | -0.38 (-0.52 to -0.24) | -4.83 (-5.82 to -3.83) | -0.14 (-0.27 to -0.01) |
| ≥$10 | -6.78 (-7.71 to -5.86) | -0.36 (-0.49 to -0.24) | -4.46 (-5.33 to -3.59) | -0.04 (-0.15 to 0.08) |
| **Urbanity** | | | | |
| Urban | -5.77 (-6.68 to -4.86) | -0.05 (-0.17 to 0.07) | -6.86 (-7.61 to -6.12) | -0.21 (-0.31 to -0.12) |
| Suburban | -7.07 (-7.75 to -6.29) | -0.26 (-0.36 to -0.17) | -4.80 (-5.43 to -4.17) | -0.19 (-0.28 to -0.11) |
| Rural | -9.30 (-10.60 to -8.00) | -0.26 (-0.42 to -0.09) | -6.29 (-7.60 to -4.99) | -0.19 (-0.36 to -0.03) |
| **Low income** | | | | |
| Yes | -8.90 (-9.81 to -7.99) | 0.03 (-0.08 to 0.15) | -6.82 (-7.56 to -6.07) | -0.17 (-0.26 to -0.07) |
| No | -7.55 (-8.16 to -6.93) | -0.55 (-0.63 to -0.47) | -4.95 (-5.46 to -4.45) | -0.06 (-0.13 to 0.00) |
| **Mail-Order** | | | | |
| Yes | -2.41 (-4.91 to 0.09) | 0.03 (-0.32 to 0.37) | -4.24 (-6.28 to -2.19) | 0.02 (-0.26 to 0.30) |
| No | -8.16 (-8.69 to -7.64) | -0.37 (-0.44 to -0.30) | -5.65 (-6.08 to -5.22) | -0.10 (-0.16 to -0.05) |

Data source: IQVIA RWD, LRX/Medical Claims, January 2011-December 2016
Abbreviations: CI, confidence interval.
[a] "Level" refers to the immediate impact of the pharmacy closure on medication adherence.
[b] "Slope" refers to the subsequent rate of change in adherence resulting from pharmacy closure.

© 2019 Qato DM et al. *JAMA Network Open*.

**eTable 7. Impact of Pharmacy Closures on β-Blocker and Oral Anticoagulant Adherence Stratified by Patient Subgroups**

## A. Impact of pharmacy closures on β-blocker adherence stratified by patient subgroups

| | Percent Change, % (95% CI) | | | |
|---|---|---|---|---|
| | All Patients | | Patients Fully Adherent at Baseline | |
| | Level[a] | Slope[b] | Level[a] | Slope[b] |
| Overall | -5.71 (-5.96 to -5.46) | -0.20 (-0.23 to -0.17) | -8.07 (-8.38 to -7.77) | -0.22 (-0.26 to -0.18) |
| Index pharmacy type | | | | |
| Chain | -6.37 (-6.90 to -5.84) | -0.23 (-0.30 to -0.16) | -8.44 (-9.07 to -7.82) | -0.27 (-0.36 to -0.19) |
| Independent | -8.01 (-8.50 to -7.56) | -0.30 (-0.36 to -0.24) | -10.70 (-11.33 to -10.07) | -0.32 (-0.40 to -0.24) |
| Mass merchandiser | -4.85 (-5.47 to -4.23) | -0.07 (-0.15 to 0.02) | -9.09 (-9.87 to -8.32) | -0.14 (-0.25 to 0.04) |
| Food | -3.75 (-4.16 to -3.34) | -0.19 (-0.24 to -0.13) | -5.22 (-5.70 to -4.75) | -0.18 (-0.25 to -0.11) |
| Mail order | | | | |
| Yes | -2.08 (-3.30 to -0.85) | -0.13 (-0.29 to 0.04) | -1.35 (-2.73 to 0.04) | -0.24 (-0.43 to -0.05) |
| No | -5.85 (-6.11 to -5.59) | -0.20 (-0.24 to -0.17) | -8.38 (-8.69 to -8.07) | -0.22 (-0.26 to -0.17) |
| Share of all prescriptions filled at index pharmacy | | | | |
| 100% | -6.67 (-7.00 to -6.34) | -0.34 (-0.438 to -0.30) | -9.53 (-9.93 to -9.12) | -0.40 (-0.45 to -0.35) |
| ≥50% to <99% | -4.37 (-4.78 to -3.96) | 0.01 (-0.05 to 0.06) | -5.95 (-6.44 to -5.47) | 0.05 (-0.02 to 0.12) |
| <50% | -4.07 (-5.07 to -3.07) | -0.04 (-0.17 to 0.10) | -4.94 (-6.17 to -3.70) | -0.02 (-0.19 to 0.15) |
| Polypharmacy (use of ≥ 5 medications) | | | | |
| Yes | -5.83 (-6.09 to -5.57) | -0.19 (-0.23 to -0.16) | -8.11 (-8.43 to -7.80) | -0.20 (-0.25 to -0.16) |
| No | -4.15 (-5.07 to -3.24) | -0.28 (-0.40 to -0.16) | -7.40 (-8.64 to -6.16) | -0.41 (-0.59 to -0.24) |
| Payment method | | | | |
| Cash | -4.19 (-5.18 to -3.19) | -0.46 (-0.59 to -0.32) | -6.27 (-7.54 to -5.00) | -0.45 (-0.63 to -0.27) |
| Medicaid | -7.47 (-8.78 to -6.15) | -0.10 (-0.27 to 0.06) | -12.04 (-13.83 to -10.25) | -0.02 (-0.25 to 0.21) |
| Medicare part D | -5.97 (-6.34 to -5.61) | -0.16 (-0.21 to -0.12) | -8.08 (-8.51 to -7.64) | -0.16 (-0.22 to -0.10) |
| Third party | -5.48 (-5.86 to -5.10) | -0.21 (-0.26 to -0.16) | -7.99 (-8.45 to -7.52) | -0.27 (-0.33 to -0.21) |
| Copayment | | | | |
| $0 to <$5 | -6.28 (-6.61 to -5.95) | -0.14 (-0.18 to -0.10) | -8.84 (-9.25 to -8.44) | -0.12 (-0.17 to -0.07) |
| ≥$5 to <$10 | -4.82 (-5.39 to -4.26) | -0.34 (-0.41 to -0.26) | -7.32 (-7.98 to -6.66) | -0.36 (-0.46 to -0.27) |
| ≥$10 | -4.86 (-5.40 to -4.32) | -0.24 (-0.31 to -0.16) | -6.67 (-7.30 to -6.03) | -0.34 (-0.43 to -0.25) |
| Community type | | | | |
| White | -5.60 (-5.89 to -5.31) | -0.21 (-0.25 to -0.18) | -7.92 (-8.27 to -7.58) | -0.25 (-0.30 to -0.20) |
| Black | -6.18 (-7.13 to -5.23) | -0.13 (-0.25 to -0.01) | -8.89 (-10.14 to -7.64) | -0.06 (-0.22 to 0.10) |
| Hispanic/Latino | -6.50 (-7.54 to -5.46) | -0.05 (-0.18 to 0.09) | -9.38 (-10.75 to -8.01) | -0.05 (-0.23 to 0.13) |
| Diverse | -5.58 (-6.28 to -4.89) | -0.25 (-0.34 to -0.16) | -7.89 (-8.73 to -7.04) | -0.17 (-0.28 to -0.06) |
| Other | -8.67 (-12.63 to -4.70) | 0.25 (-0.26 to 0.76) | -10.60 (-15.55 to -5.64) | 0.10 (-0.55 to 0.75) |
| Urban-Rural | | | | |
| Urban | -5.02 (-5.45 to -4.59) | -0.14 (-0.20 to -0.08) | -6.95 (-7.46 to -6.44) | -0.12 (-0.19 to -0.05) |
| Suburban | -5.33 (-5.69 to -4.98) | -0.16 (-0.20 to -0.11) | -7.77 (-8.19 to -7.34) | -0.16 (-0.22 to -0.11) |
| Rural | -8.04 (-8.65 to -7.43) | -0.43 (-0.50 to -0.35) | -10.83 (-11.59 to -10.08) | -0.54 (-0.64 to -0.44) |
| Low income | | | | |
| Yes | -6.68 (-7.15 to -6.21) | -0.12 (-0.18 to -0.06) | -9.44 (-10.03 to -8.84) | -0.07 (-0.14 to 0.01) |
| No | -5.30 (-5.60 to -5.01) | -0.23 (-0.27 to -0.19) | -7.55 (-7.90 to -7.20) | -0.27 (-0.32 to -0.22) |
| Pharmacy Density | | | | |
| Quintile 1 | -7.98 (-8.58 to -7.37) | -0.43 (-0.51 to -0.36) | -10.81 (-11.56 to -10.06) | -0.53 (-0.63 to -0.43) |
| Quintile 5 | -4.90 (-5.39 to -4.42) | -0.17 (-0.23 to -0.11) | -6.65 (-7.23 to -6.07) | -0.18 (-0.26 to -0.11) |

Abbreviations: CI, confidence interval.

© 2019 Qato DM et al. *JAMA Network Open*.

**B. Impact of pharmacy closures on oral anticoagulant adherence stratified by patient subgroups**

| | Percent Change, % (95% CI) | | | |
|---|---|---|---|---|
| | All Patients | | Patients Fully Adherent at Baseline | |
| | Level[a] | Slope[b] | Level[a] | Slope[b] |
| Overall | -5.63 (-6.24 to -5.01) | -0.15 (-0.23 to -0.07) | -8.37 (-9.21 to -7.54) | -0.20 (-0.31 to -0.09) |
| Index pharmacy type | | | | |
| Chain | -5.48 (-6.84 to -4.13) | -0.41 (-0.58 to -0.23) | -7.06 (-8.85 to -5.27) | -0.56 (-0.80 to -0.32) |
| Independent | -7.31 (-8.46 to -6.16) | -0.24 (-0.38 to -0.09) | -10.30 (-11.93 to -8.66) | -0.31 (-0.52 to -0.10) |
| Mass merchandiser | -5.70 (-7.22 to -4.17) | 0.01 (-0.18 to 0.21) | -10.88 (-13.08 to -8.69) | 0.07 (-0.21 to 0.35) |
| Food | -4.28 (-5.30 to -3.26) | -0.03 (-0.17 to 0.10) | -6.36 (-7.69 to -5.03) | -0.07 (-0.25 to 0.12) |
| Mail order | | | | |
| Yes | -0.79 (-3.61 to 2.03) | -0.12 (-0.51 to 0.27) | 0.09 (-3.27 to 3.45) | -0.10 (-0.59 to 0.39) |
| No | -5.83 (-6.46 to -5.21) | -0.15 (-0.23 to -0.07) | -8.86 (-9.72 to -8.00) | -0.21 (-0.32 to -0.09) |
| Share of all prescriptions filled at index pharmacy | | | | |
| 100% | -6.98 (-7.79 to -6.17) | -0.30 (-0.40 to -0.19) | -10.80 (-11.15 to -8.91) | -0.39 (-0.54 to -0.24) |
| ≥50% to <99% | -4.09 (-5.09 to -3.09) | 0.06 (-0.07 to 0.18) | -6.36 (-7.71 to -5.02) | 0.05 (-0.13 to 0.23) |
| <50% | -2.52 (-4.92 to -0.12) | -0.08 (-0.41 to 0.24) | -3.97 (-7.16 to -0.78) | -0.04 (-0.489 to 0.41) |
| Polypharmacy (use of ≥ 5 medications) | | | | |
| Yes | -5.74 (-6.37 to -5.11) | -0.14 (-0.22 to -0.06) | -8.46 (-9.31 to -7.61) | -0.18 (-0.29 to -0.07) |
| No | -3.08 (-5.52 to -0.64) | -0.35 (-0.68 to -0.02) | -5.93 (-10.12 to -1.74) | -0.80 (-1.37 to -0.23) |
| Payment method | | | | |
| Cash | -5.05 (-7.72 to -2.39) | -0.37 (-0.73 to -0.02) | -9.12 (-13.04 to -5.21) | -0.21 (-0.77 to 0.34) |
| Medicaid | -7.88 (-11.81 to -3.95) | -0.31 (-0.79 to 0.18) | -12.96 (-18.94 to -6.98) | -0.29 (-1.03 to 0.45) |
| Medicare part D | -5.91 (-6.75 to -5.06) | -0.12 (-0.23 to -0.02) | -8.92 (-10.06 to -7.78) | -0.13 (-0.28 to 0.02) |
| Third party | -5.19 (-6.15 to -4.24) | -0.15 (-0.27 to -0.02) | -7.24 (-8.55 to -5.92) | -0.30 (-0.48 to -0.13) |
| Copayment | | | | |
| $0 to <$5 | -6.06 (-6.91 to -5.21) | -0.12 (-0.22 to -0.01) | -9.23 (-10.43 to -8.04) | -0.12 (-0.28 to 0.03) |
| ≥$5 to <$10 | -5.98 (-7.30 to -4.66) | -0.15 (-0.32 to 0.02) | -7.84 (-9.62 to -6.06) | -0.15 (-0.39 to 0.09) |
| ≥$10 | -4.58 (-5.74 to -3.42) | -0.22 (-0.37 to -0.06) | -7.21 (-8.74 to -5.68) | -0.38 (-0.59 to -0.16) |
| Community type | | | | |
| White | -5.55 (-6.24 to -4.86) | -0.15 (-0.24 to -0.07) | -8.41 (-9.34 to -7.47) | -0.21 (-0.34 to -0.09) |
| Black | -6.39 (-9.21 to -3.57) | -0.07 (-0.41 to 0.28) | -9.45 (-13.58 to -5.32) | 0.13 (-0.39 to 0.65) |
| Hispanic/Latino | -3.48 (-6.31 to -0.64) | -0.41 (-0.78 to -0.05) | -6.28 (-9.88 to -2.69) | -0.62 (-1.14 to -0.11) |
| Diverse | -6.73 (-8.55 to -4.92) | -0.04 (-0.28 to 0.20) | -8.47 (-10.99 to -5.94) | -0.09 (-0.42 to 0.25) |
| Other | -4.97 (-13.24 to 3.30) | -0.16 (-1.28 to 0.95) | -7.76 (-16.20 to 0.67) | -0.23 (-1.53 to 1.07) |
| Urbanity | | | | |
| Urban | -5.10 (-6.19 to -4.01) | -0.11 (-0.25 to -0.03) | -7.17 (-9.63 to -6.70) | -0.11 (-0.31 to -0.09) |
| Suburban | -4.74 (-5.60 to -3.88) | -0.14 (-0.25 to -0.03) | -6.89 (-8.06 to -5.72) | -0.22 (-0.38 to -0.06) |
| Rural | -8.89 (-10.32 to -7.45) | -0.24 (-0.42 to -0.07) | -12.52 (-14.51 to -10.5) | -0.29 (-0.55 to -0.04) |
| Low income | | | | |
| Yes | -6.83 (-8.06 to -5.61) | -0.01 (-0.16 to 0.14) | -9.67 (-11.36 to -7.98) | 0.01 (-0.21 to 0.23) |
| No | -5.21 (-5.91 to -4.50) | -0.20 (-0.29 to -0.11) | -7.93 (-8.89 to -6.97) | -0.27 (-0.40 to -0.14) |
| Pharmacy Density | | | | |
| Quintile 1 | -7.70 (-9.19 to -6.27) | -0.36 (-0.54 to -0.19) | -11.69 (-13.70 to -9.68) | -0.38 (-0.63 to -0.12) |
| Quintile 5 | -4.91 (-6.08 to -3.74) | -0.13 (-0.28 to 0.02) | -7.36 (-8.95 to -5.78) | 0.10 (-0.17 to 0.36) |

Abbreviations: CI, confidence interval.

**eTable 8.** Effect of pharmacy closures on statin adherence stratified by patient subgroups overall and among patients fully adherent at baseline

| | Absolute Percent Change, % (95% CI) | | | |
|---|---|---|---|---|
| | Overall | | Fully Adherent at Baseline | |
| | Level[a] | Slope[b] | Level[a] | Slope[b] |
| Overall | -5.90 (-6.12 to -5.69) | -0.15 (-0.18 to -0.13) | -8.57 (-8.84 to -8.30) | -0.18 (-0.22 to -0.15) |
| Expected refill date after last statin fill during baseline | | | | |
| >30 days before closure | -0.22 (-0.55 to -0.11) | -0.01 (-0.06 to 0.05) | -1.75 (-2.92 to -0.58) | -0.23 (-0.29 to -0.06) |
| 15-30 days before closure | -6.91 (-8.13 to -5.70) | -0.00 (-0.14 to 0.15) | -8.41 (-10.41 to -6.40) | -0.16 (-0.39 to 0.07) |
| 0-14 days before closure | -11.80 (-12.70 to -10.89) | 0.31 (0.21 to 0.42) | -14.44 (-15.69 to -13.18) | 0.42 (0.29 to 0.55) |
| 1-14 days after closure | -14.67 (-15.18 to -14.16) | 0.43 (0.37 to 0.49) | -15.72 (-16.31 to -15.13) | 0.48 (0.41 to 0.54) |
| 15-30 days after closure | -6.57 (-6.95 to -6.19) | -0.39 (-0.45 to -0.34) | -7.40 (-7.79 to -7.00) | -0.35 (-0.41 to -0.28) |
| >30 days after closure | -1.91 (-2.24 to -1.58) | -0.80 (-0.85 to -0.74) | -2.63 (-2.96 to -2.31) | -0.82 (-0.88 to -0.75) |

Data source: IQVIA RWD, LRX/Medical Claims, January 2011-December 2016

**eFigure 1. Cohort Selection for β-Blocker and Oral Anticoagulant Users**

**A. Cohort selection for β-blocker users**

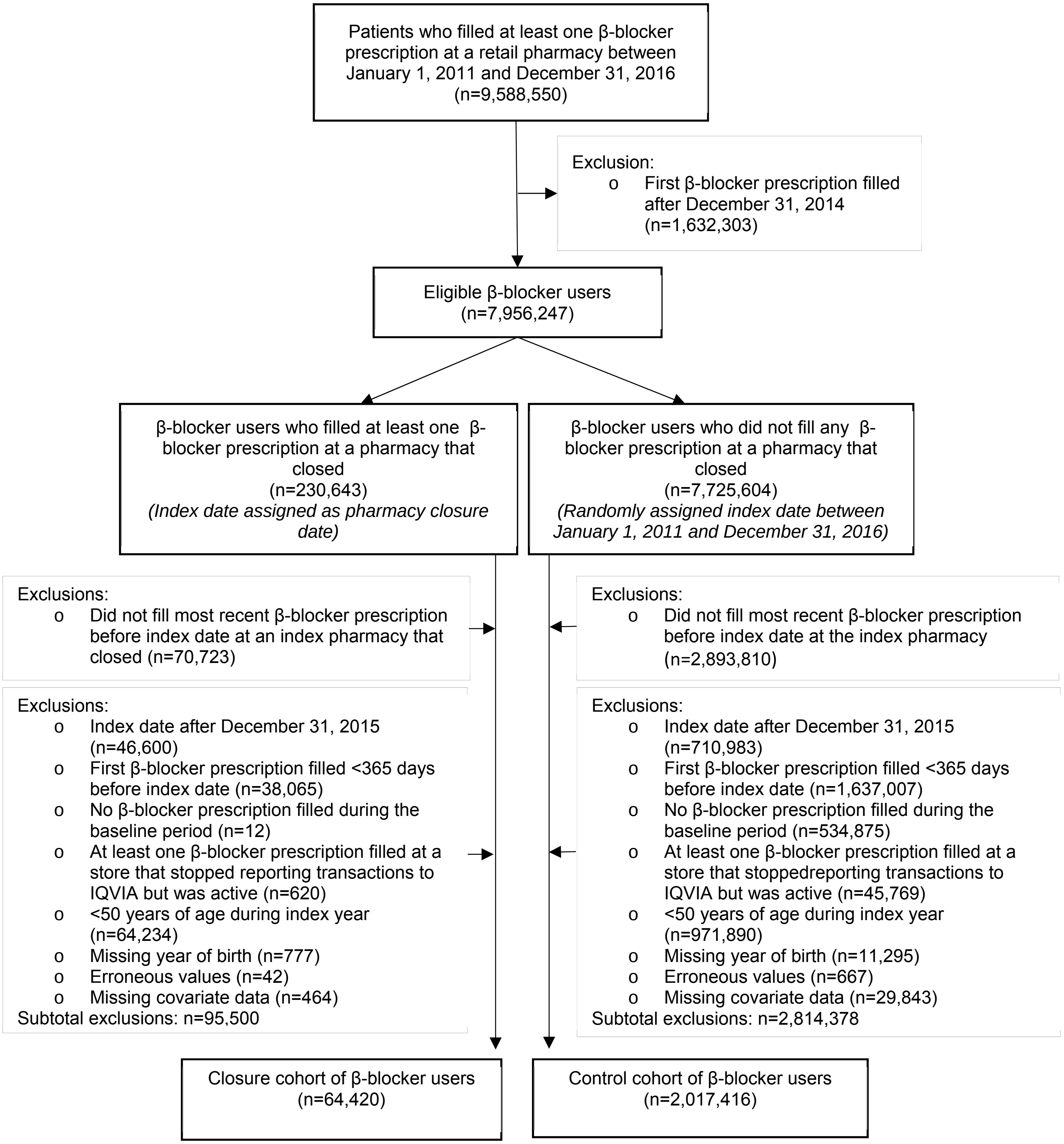


© 2019 Qato DM et al. *JAMA Network Open*.

**B. Cohort selection for oral anticoagulant users**

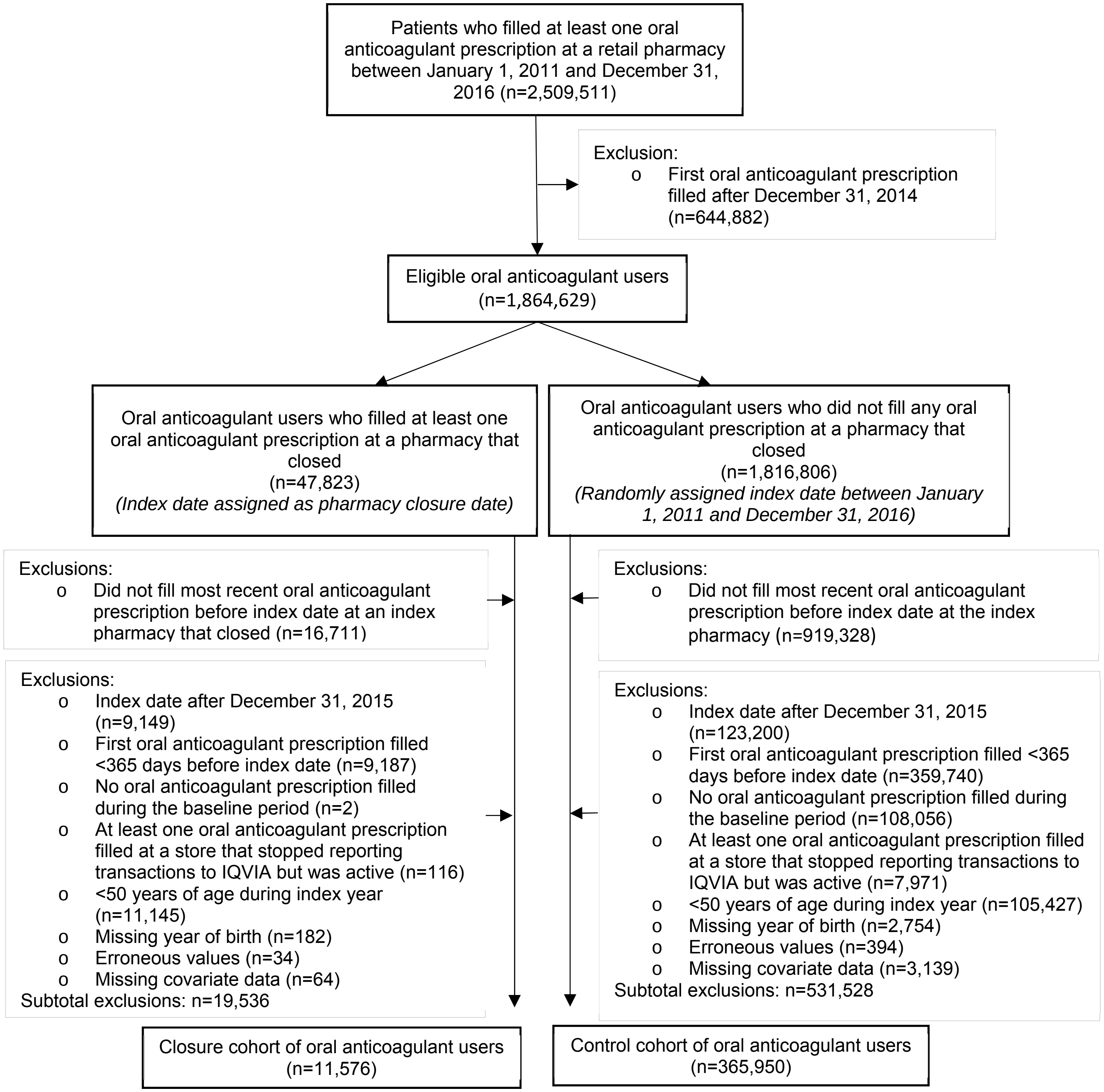


© 2019 Qato DM et al. *JAMA Network Open*.

**eFigure 2. Distribution of proportion of days covered (PDC) during baseline and follow-up**

Closure cohort | Non-closure cohort

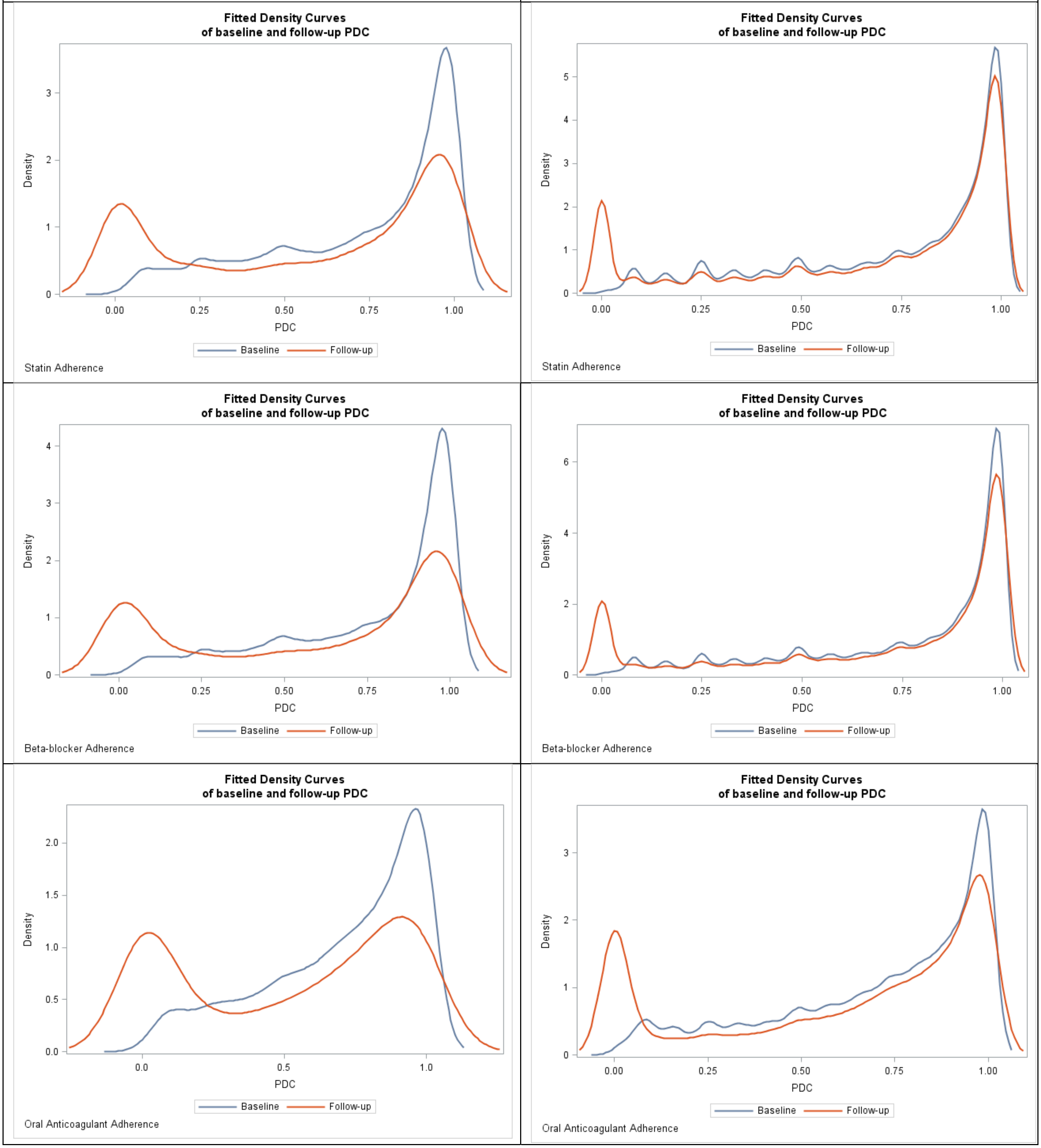


© 2019 Qato DM et al. *JAMA Network Open*.
